# Associations between illicit drug use in early adulthood and mortality: Findings from a National Birth Cohort

**DOI:** 10.1016/j.ypmed.2022.107058

**Published:** 2022-06

**Authors:** James White

**Affiliations:** Centre for Trials Research, Cardiff University, 4th floor, Neuadd Meirionnydd, Heath Park, Cardiff CF14 4YS, UK

## Abstract

Illicit drug use is known to be associated with premature mortality. Whether exposure to socioeconomic disadvantage and mental health problems in childhood help to explain this association, is unclear. We analysed data from 11,250 participants in the 1970 British Birth Cohort study. At 10-years of age, socioeconomic disadvantage (parental socioeconomic position, material disadvantage, family disruption) and mental health problems with antisocial behaviour, attention, and anxiety were reported by mothers and teachers. At 30-years of age, study members provided information on their illicit drug use, exposure to socioeconomic disadvantage and mental health problems. At 30-years, 19.2% of participants had used an illicit drug in the past year. Mortality was elevated for eight of the twelve drugs assessed. Family disruption, maternal, and teacher assessments of antisocial behaviour at 10-years were associated with illicit drug use at 30-years. There was, however, very little change in these associations when exposure to childhood socioeconomic disadvantage (% change in hazard ratios [HR] 0–10%) or mental health problems (0.4–11.9%) were added to the sex-adjusted model. Adding exposure to socioeconomic disadvantage (0.8–38.9%) and mental health problems (31.7–74.1%) in adulthood to the sex-adjusted model resulted in marked attenuation in HRs for all drugs. These findings imply that interventions which provide opportunities for education, employment and access to effective mental health treatments in early adulthood may help to reduce mortality among drug users.

## Highlights

• Illicit drug use was associated with premature mortality.
• Family disruption in childhood was associated with adult drug use.
• Mental health problems in early adulthood were associated with opiate use.
• Premature mortality linked to drug use was not explained by childhood experiences.
• Adult disadvantage and mental health explained mortality linked to drug use.

## 1 Introduction

A series of studies have documented that people with a drug dependency experience an increased risk of mortality (Degenhardt et al., 2011; Eaton et al., 2013; Fontanella et al., 2021; Lopez-Quintero et al., 2015). In the few cohorts examining use rather than dependency, lifetime heroin and cocaine (Walker et al., 2017), and regular opioid use (Degenhardt et al., 2011), have been linked to an elevated mortality-risk. Lifetime cannabis use was not associated with elevated mortality in one study (Sun et al., 2020), but another reported an increased risk emerged at use >50 times across a lifetime (Manrique-Garcia et al., 2016). There are even fewer reports on mortality-risks associated with the use of other drugs such as amphetamines (Singleton et al., 2009) and hallucinogens (Walker et al., 2017).

Aside from the risks of overdose, explanations of why illicit drug use is associated with premature mortality include the potential confounding role of early life exposure to socioeconomic disadvantage and mental health problems. An association between exposure to socioeconomic disadvantage in childhood with subsequent illicit drug use (Daniel et al., 2009) and premature mortality (Galobardes et al., 2004) has been documented across a number of cohorts. Early life anxiety and behavioural disorders have also been associated with subsequent drug use (Conway et al., 2016; Lynskey and Fergusson, 1995), and an increased risk of mortality (Plana-Ripoll et al., 2019). The mechanisms linking illicit drug use with mortality are unclear. The candidate explanations include a ‘self-medication’ hypothesis (Khantzian, 1985), where drugs are used to cope with the effects of a childhood characterised by economic disadvantage, or a psychiatric disorder. Associations with mortality may also arise through an increased susceptibility to illness caused directly by disadvantage in childhood (Lundberg, 1993), psychiatric medication shortening lifespan (Maslej et al., 2017; Ralph and Espinet, 2018), or a combination of these mechanisms.

To examine the extent socioeconomic disadvantage and mental health problems in childhood may ‘explain’ the mortality risk associated with drug use - that is the degree to which it may account for the variance in this association - can be tested by adjusting the relationship between mortality and illicit drugs for these characteristics. In the only three reports of which we are aware, exposure to socioeconomic disadvantage in adulthood did not offer much explanatory power, but there was marked attenuation when negative mood was added to models (Muhuri and Gfroerer, 2011; Qureshi et al., 2014; Walker et al., 2017). However, importantly, these studies did not assess exposure to socioeconomic disadvantage and mental health problems in childhood. This is possible in the 1970 British Birth Cohort Study, an ongoing prospective cohort with extensive assessments of illicit drug use, socioeconomic disadvantage and mental health problems in childhood and later adult life, and mortality. We hypothesized that socioeconomic disadvantage and mental health problems in childhood would explain the association between illicit drug use and mortality.

## 2 Methods

### 2.1 Study population

The 1970 British Birth Cohort Study is an ongoing geographically-representative, prospective birth cohort of children born in Great Britain. A detailed description of attrition is provided elsewhere (Plewis et al., 2004). The main reason for loss to follow-up was members moving and not subsequently being traced and refusal rates were relatively low (7.3%). The target sample consisted of all 17,287 babies born (including stillbirths) in England, Scotland and Wales between the 5th and 11th April 1970. Data were initially collected by the midwifes present at the birth and from clinical records. A total of 16,571 babies were enrolled and have been followed up on ten occasions. Ethical approval was given by the London Central Research Ethics Committee. Written informed consent was given by parents of study participants before the start of data collection. We adhered to the guidelines for STrengthening the Reporting of OBservational studies in Epidemiology (STROBE) in the reporting in this manuscript (von Elm et al., 2007).

### 2.2 Outcome variable

The outcome is all-cause mortality. Members were followed up until 31st December 2014 (age 44). A total of 880 deaths occurred. Of these, 751 (85.3%) occurred before the assessment of illicit drug use so were excluded. Of the 751 deaths excluded, 567 (75.5%) were stillborn or neonatal deaths (defined as within 28 days of birth). Vital status was derived from official death certificates from the National Health Service Central Register, or from relatives and friends during fieldwork and cohort maintenance work by telephone, letter and e-mail (<1% of deaths). In only 40 cases the date of death was unknown.

### 2.3 Exposure variables

The exposures of interest were illicit drug use in adulthood assessed at 30 years of age. Study members reported on the lifetime use of cannabis, stimulants: (ecstasy, amphetamines, cocaine, crack), hallucinogens: (lysergic acid diethylamide (LSD), magic mushrooms), opiates (heroin, methadone), poppers, temazepam, and ketamine. Street names of drugs were also provided. Response options were: never, taken but not in the past year, and taken in the past year.

### 2.4 Covariates

Covariates were identified a priori. At 10-years, exposure to socioeconomic disadvantage was based on mother's and father's occupation provided at interview and coded using the Registrar General's classification system (General, 1980). Material disadvantage was assessed using information provided by parents with one point was assigned for the presence of each of the following: the family did not own their home, the household was over-crowded (one or more person per room), the household had no access or had to share access to one or more of three basic amenities (hot water, use of a bathroom, and lavatory), or they had received state benefits because of financial need in the last year (i.e. child benefit and retirement pensions were excluded). Higher scores indicated greater material disadvantage (Schoon et al., 2003). Family disruption was a binary variable calculated using parents reports on any occurrence of parental divorce, separation or death by 10-years of age.

Childhood mental health was based on the 19-item maternal reported Rutter Parent Scale (Rutter et al., 1970), and teacher reported 53-item Social Development Scale, derived from the Conners Teacher Rating Scale (Conners, 1969) and the Rutter Teaching Scale (Rutter, 1967). Principal components analysis (PCA) by Gale, found maternal ratings could be organised into three groups: anti-social behaviour, anxiety and attention problems (Gale et al., 2009). We repeated this analysis and the scree slope suggested the presence of the same three factors after oblique rotation which accounted for 42.3% of the total variance. PCA on the teacher reported Social Development Scale also suggested a three-factor solution accounting for 44.4% of the total variance, with items loading on the same grouping of symptoms/ behaviours (White et al., 2012). For all six factors, composite scores were computed using regression then standardized.

Maternal psychological morbidity were reported at 10-years using Rutter's Malaise Inventory (Rutter et al., 1970). It's based on the Cornell medical index and consists of a 15-question scale for psychological symptoms and an 8-question subscale for somatic symptoms. Scores were totalled across items then standardized.

At 30-years, exposure to socioeconomic disadvantage was based on responses to enquiries about current occupation using the Registrar General's classification system (General, 1980) and achieved educational qualifications. Mental health problems were ascertained using self-reports on treatment for a psychiatric problem since 16 years of age and psychological morbidity based on a score of ≥7 on the Rutter Malaise Inventory (Rodgers et al., 1999). All participants, apart from lifelong teetotallers, were asked to complete questions on possible problems with alcohol using the cutting down, being annoyed by criticism, feeling guilty, and eye-openers (CAGE) questionnaire (Ewing, 1984). Daily smokers were those smoking at least one cigarette every day in the past week.

### 2.5 Statistical analyses

We imputed all missing exposure, outcome and covariate data using multiple imputation by chained equations which included all variables in the prediction model to generate 20 datasets. We included the Nelson–Aalen estimate of the cumulative hazard of survival time to the imputation model to increase statistical power (White and Royston, 2009). We then deleted any imputed outcome data before analysis as this method has been found to produce less variable point estimates and narrower confidence intervals than using imputed outcome data (Von Hippel, 2007). Our main results are based on analyses with the imputed sample as they offered greater precision (*n* = 11,250 participants; 5787 women).

To test differences in childhood and adult characteristics by illicit drug use we used a logistic regression model. We used Cox proportional hazards models to compute hazard ratios (HR) with accompanying 95% confidence intervals (95% CI) to summarise the association between illicit drug use and mortality. We ascertained that the proportional hazards assumption had not been violated in each cohort by inspecting the Schoenfeld residuals. We did not find interactions between illicit drug use and sex in the association with mortality, so data were aggregated, and sex adjusted. We adjusted HRs for sex (the basic model), then added exposure to socioeconomic disadvantage in childhood, childhood antisocial behaviour, attention and anxiety, maternal psychological morbidity, then socioeconomic disadvantage, psychological morbidity plus treatment for a psychiatric problem, and finally alcohol problems and smoking in adulthood. We summarised any changes in hazard ratios after adjusting for each set of covariates using the formula: [HR _basic model_ − 1] - [HR _adjusted model_-1] / [HR _basic model_-1] x 100% (Batty et al., 2006). We did not adjust for the potential inflation of the type I error rate from multiple testing as our analyses were exploratory rather than confirmatory (Li et al., 2017). We investigated potential collinearity between exposures and covariates by estimating the variance inflation factor.

We conducted a number of sensitivity analyses. To examine the impact of missing data we re-ran the analyses in those with complete data. As the effects of socioeconomic disadvantage and mental health problems may interact, we then modelled interactions between these variables in childhood and adulthood. Then, to explore the effect of opiate use in driving associations we conducted a sub-group analysis examining drug use-mortality associations after excluding opiate users. All analyses were performed using Stata version 16.1 and R Studio 1.4.1717.

## 3 Results

eFig. 1 shows a flow diagram of participation and missing data. Of the 16,571 individuals originally recruited, 11,250 (67.9%) provided information illicit drug use plus covariates. Missing data per variable ranged from 0 to 46.0%. There were 4032 participants with no missing data which made up the complete case sample. People with missing data were more likely to have parent with an unskilled occupation, mother with a higher psychological morbidity score, have no qualifications, but have experienced less family disruption, fewer problems with antisocial behaviour, attention, and anxiety (maternal rating only), and likely to screen positive for psychological morbidity, an alcohol problem, or smoke daily (all *P*-values <0.05; results available on request).

### 3.1 Association of Illicit Drug use with socioeconomic disadvantage and mental health

**Fig. 1:**
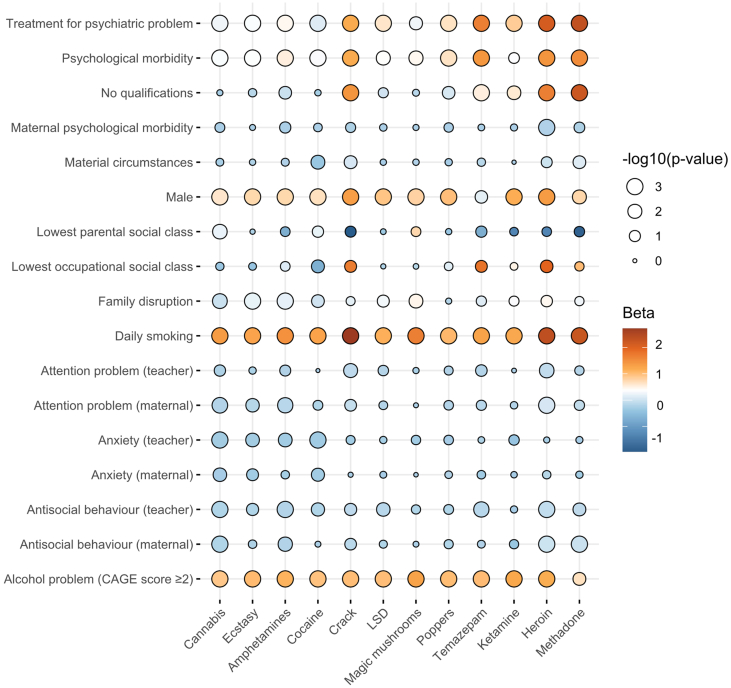
Balloon plot illustrating childhood and adult characteristic-illicit drug association pairs (*n* = 11,250).

At 30-years, 19.2% of participants had used an illicit drug in the past year. Cannabis was the most used drug (16.9%), followed by cocaine (5.8%), ecstasy (4.3%), and amphetamines (4.2%). Figure 1 shows that in childhood, exposure to family disruption was associated with an increased risk of adult stimulant and hallucinogen use. Maternal and teacher ratings of antisocial behaviour were associated with later stimulant and opiate use, and anxiety ratings with a lower risk of stimulant use. Maternal psychological morbidity was essentially unrelated to drug use. Of the adult characteristics, being male, a daily smoker or having an alcohol problem, were most consistently related to drug use. There was also a clustering crack and opiate use among people who had been treated for a psychiatric condition, a psychological morbidity, and those without any qualifications. Correlation coefficients for the association between exposures and covariates ranged from 0.0 (cocaine use and attention problems) to 2.95 (crack and daily smoking; Supplementary tables 1 and 2) and the mean variance inflation factor was 1.50, suggesting limited evidence of collinearity.

### 3.2 Association of Illicit Drug use with mortality

There were 129 deaths over a median follow-up of 7.2 years (interquartile range [IQR], 3.5 to 10.1). Table 1 shows that relative to people who had not used in the past year, those who had used amphetamines, poppers, temazepam, ketamine, crack, heroin and methadone, were at an increased risk of premature mortality. Adjustment for childhood socioeconomic disadvantage reduced the strength of the illicit drug use and mortality association by 0 to 10%. Table 1 and Supplementary Figs. 1 and 2 show the inclusion of maternal and teacher assessments of childhood mental health reduced the strength of the association by between 0.42 and 11.9%, with the largest reductions for crack and opiate use. Table 2 shows the addition of socioeconomic disadvantage in adulthood reduced the illicit drug use-mortality association by 0.83 to 38.9% (crack: 28.3%, heroin: 30.2%, methadone: 23.5%), mental health by 31.7 to 74.1%, and alcohol problems and daily smoking by 26.1 to 100%.

**Table 1:** Association of illicit drug use in the past year with all-cause mortality after adjusting for childhood characteristics (***n*** = 11,250).

	Hazard ratio (95% confidence interval)							
	% (n)	Sex-adjusted	Adjusted for sex plus childhood socioeconomic disadvantage a	% change b	Adjusted for sex plus childhood mental health ^a^	% change b	Adjusted for sex plus maternal psychological morbidity	% change b
Any drug in past year	19.2 (2162)	1.42 (0.95, 2.11)	1.42 (0.95, 2.12)	0	1.40 (0.94, 2.10)	4.76	1.42 (0.95, 2.12)	0
Cannabis	16.9 (1901)	1.27 (0.83, 1.95)	1.27 (0.83, 1.96)	0	1.25 (0.82, 1.92)	7.41	1.27 (0.83, 1.96)	0
Ecstasy	4.3 (484)	1.49 (0.75, 2.95)	1.48 (0.75, 2.92)	2.04	1.50 (0.76, 2.97)	2.04	1.49 (0.76, 2.95)	0
Amphetamines	4.2 (473)	2.17 (1.19, 3.95)	2.14 (1.17, 3.89)	2.56	2.14 (1.17, 3.91)	2.56	2.17 (1.19, 3.94)	0
LSD	0.9 (101)	2.20 (0.70, 6.94)	2.08 (0.66, 6.58)	10.00	2.18 (0.69, 6.88)	1.67	2.20 (0.70, 6.92)	0
Magic mushrooms	0.8 (90)	1.82 (0.45, 7.37)	1.76 (0.43, 7.17)	7.32	1.86 (0.46, 7.54)	−4.88	1.82 (0.45, 7.39)	0
Poppers	1.7 (191)	3.39 (1.65, 6.97)	3.43 (1.67, 7.07)	−1.67	3.38 (1.64, 6.97)	0.42	3.38 (1.65, 6.96)	0.42
Cocaine	5.8 (653)	1.67 (0.94, 2.97)	1.67 (0.94, 2.99)	0	1.69 (0.95, 3.02)	−2.99	1.67 (0.94, 2.99)	0
Temazepam	1.0 (113)	4.16 (1.70, 10.19)	4.11 (1.68, 10.09)	1.58	4.08 (1.67, 10.00)	2.53	4.15 (1.69, 10.14)	0.32
Ketamine	0.3 (34)	4.12 (1.02, 16.73)	3.92 (0.96, 15.97)	6.41	4.33 (1.06, 17.72)	−6.73	4.13 (1.02, 16.75)	−0.32
Crack	0.5 (56)	6.12 (2.25, 16.61)	5.88 (2.15, 16.05)	4.69	5.64 (2.05, 15.46)	9.38	6.07 (2.23, 16.52)	0.98
Heroin	0.4 (45)	10.84 (4.74, 24.79)	10.92 (4.75, 25.15)	−0.81	9.67 (4.14, 22.55)	11.89	10.76 (4.70, 24.64)	0.81
Methadone	0.3 (34)	13.24 (5.39, 32.53)	13.14 (5.28, 32.68)	0.82	12.06 (4.84, 30.00)	9.64	13.16 (5.35, 32.35)	0.65

LSD = lysergic acid diethylamide.

a Childhood socioeconomic disadvantage comprises paternal (maternal if paternal missing) registrar General's classification, material circumstances (sum of renting, household overcrowding (+1 person per room), receipt of state benefits, and no or shared access of either a bathroom, lavatory or hot water), and family disruption (any parental divorce, separation or death); childhood mental health assessed antisocial behaviour, anxiety, and attention problems using the Rutter parental ‘A' scale of behaviour disorder (maternal report) and the social development scale (teacher report).

b Based on a comparison of the sex-adjusted HR with that for the covariate-adjusted HR using the formulae: ([HR _sex-adjusted_ - 1]/ [HR _covariate-adjusted_ - 1]/[HR _sex-adjusted_ - 1]) / 100%.

**Table 2:** Association of illicit drug use in the past year with all-cause mortality after adjusting for adult characteristics (*n* = 11,250).

	Hazard ratio (95% confidence interval)						
	Sex-adjusted	Adjusted for sex plus adult socioeconomic disadvantage a	% change b	Adjusted for sex plus adult mental health c	% change	Adjusted for sex plus adult alcohol problems and daily smoking d	% change
Any drug in past year	1.42 (0.95, 2.11)	1.38 (0.92, 2.07)	9.52	1.18 (0.79, 1.78)	57.14	1.08 (0.70, 1.65)	80.95
Cannabis	1.27 (0.83, 1.95)	1.24 (0.80, 1.91)	11.11	1.07 (0.69, 1.65)	74.07	0.95 (0.61, 1.49)	100
Ecstasy	1.49 (0.75, 2.95)	1.53 (0.77, 3.03)	8.16	1.23 (0.62, 2.45)	53.06	1.11 (0.55, 2.22)	77.55
Amphetamines	2.17 (1.19, 3.95)	2.10 (1.15, 3.84)	5.98	1.77 (0.96, 3.24)	34.19	1.61 (0.86, 2.98)	47.86
LSD	2.20 (0.70, 6.94)	2.19 (0.69, 6.91)	0.83	1.74 (0.55, 5.51)	38.33	1.68 (0.53, 5.33)	43.33
Magic mushrooms	1.82 (0.45, 7.37)	1.86 (0.46, 7.54)	4.88	1.49 (0.37, 6.04)	40.24	1.26 (0.31, 5.16)	68.29
Poppers	3.39 (1.65, 6.97)	3.36 (1.63, 6.93)	1.26	2.60 (1.25, 5.39)	33.05	2.63 (1.27, 5.46)	31.80
Cocaine	1.67 (0.94, 2.97)	1.76 (0.98, 3.14)	13.43	1.43 (0.80, 2.57)	35.82	1.27 (0.70, 2.30)	59.70
Temazepam	4.16 (1.70, 10.19)	2.93 (1.07, 7.99)	38.92	2.57 (1.03, 6.42)	50.32	3.04 (1.23, 7.53)	35.44
Ketamine	4.12 (1.02, 16.73)	3.74 (0.92, 15.30)	12.18	3.13 (0.77, 12.79)	31.73	2.97 (0.73, 12.14)	36.86
Crack	6.12 (2.25, 16.61)	4.67 (1.68, 12.95)	28.32	3.92 (1.42, 10.87)	42.97	4.40 (1.60, 12.13)	33.59
Heroin	10.84 (4.74, 24.79)	7.87 (3.32, 18.67)	30.18	6.38 (2.70, 15.07)	45.33	7.66 (3.29, 17.85)	32.32
Methadone	13.24 (5.39, 32.53)	10.36 (4.06, 26.41)	23.53	7.52 (2.97, 19.09)	46.73	10.04 (4.03, 25.02)	26.14

LSD = lysergic acid diethylamide.

a Adult socioeconomic disadvantage assessed at 30-years using the registrar General's classification and whether study members had any qualifications.

b Based on a comparison of the sex-adjusted HR with that for the covariate-adjusted HR using the formulae: ([HR _sex-adjusted_ - 1]/ [HR _covariate-adjusted_ - 1]/[HR _sex-adjusted_ - 1]) / 100%.

c Adult mental health comprises psychiatric morbidity (malaise score ≥ 7) and having seen specialist for psychiatric problem.

d Alcohol problems assessed as a CAGE score ≥ 2.

### 3.3 Sensitivity analyses

The results using the complete case sample were not materially different to those derived using imputed data (see Supplementary Table 3). As there were no participants in the complete case sample who used a hallucinogen, temazepam, crack or opiate in the past year who later died we could not generate estimates for these exposures. Modelling the interaction between socioeconomic disadvantage and poor mental health had a small additional impact on associations (Supplementary Table 4). Adding interactions in adulthood further reduced HRs for crack and opiates from around 30% to over 50% but had little impact on associations for other drugs. Excluding opiate users attenuated all of the drug use-mortality associations (Supplementary Table 5).

Notes: Size of the circle is proportional to the -log10 *P*-value (3 is equivalent to *p* < 0.001; 2 *p* = 0.01; 1 *p* = 0.10, 1 = 0). Colour indicates the beta value where dark orange = positive coefficient; blue = negative coefficient.

## 4 Discussion

The purpose of these analyses was to examine the extent to which exposure to socioeconomic disadvantage and poor mental health in childhood accounted for variance in associations between illicit drug use and mortality. We did not find support for the hypothesis that exposure to socioeconomic disadvantage and mental health in childhood explained the association between drug use and mortality. We did, however, find that exposure to socioeconomic disadvantage in later adult life appeared to offer more explanatory power, accounting for around a quarter, and mental health around a half of the elevated mortality risk associated with temazepam, crack and opiate use.

As the first study to examine the explanatory power of exposure to socioeconomic disadvantage and mental health in childhood in the relation of illicit drug use in early adult life with mortality, our study is unusual. To our knowledge, only data from the 1991 National Health Interview Survey (Muhuri and Gfroerer, 2011; Walker et al., 2017) and the third National Health and Nutrition Examination Survey (Qureshi et al., 2014) have investigated whether socioeconomic disadvantage and mental health might explain the illicit-drug use-mortality relation. In these studies, adjustment for socioeconomic position and negative mood in assessed in adulthood appears to have had a less pronounced impact on associations than we observed. The effect of controlling for mental health problems may have been more pronounced in our study because we used a validated measure of psychological morbidity, rather than a single item measure (Muhuri and Gfroerer, 2011; Walker et al., 2017).

Illicit drug use in early adulthood was associated with excess mortality. Whilst there is ample evidence that drug dependence is associated with elevated mortality risk (Eaton et al., 2013; Lundin et al., 2016), the few population-based samples which have investigated mortality-risk among drug users have only examined cannabis, stimulants and opiate use. In agreement with these studies (Muhuri and Gfroerer, 2011; Walker et al., 2017) we found that whilst risks were elevated for eight of the twelve drugs assessed, mortality risk was only consistently elevated for users of poppers, crack and opiate users across all analyses. Consistent with studies linking adverse childhood experiences with drug use disorders in adulthood (Giordano et al., 2014), we found family disruption was associated with an increased risk of stimulant and hallucinogen use. We also replicated findings from the smaller Christchurch Health and Development Study, which found parent and teacher ratings of conduct problems at 8-years of age were associated with drug use in mid-adolescence (Lynskey and Fergusson, 1995). Our analysis extends the results from this study and other birth cohorts reporting similar results (Kretschmer et al., 2014) by showing that antisocial behaviour at 10-years of age predicted illicit drug 20-years later in early adulthood.

This study has a number of strengths, including the assessments of a wide range of illicit drugs, socioeconomic disadvantage and mental health problems in childhood and later adult life, as well as traditional risk factors for premature mortality. It is not, however, without its shortcomings. First, is loss to follow-up. If the pattern of missing data was one where both illicit drug use and death before the follow-up in adulthood was more common in cohort members experiencing childhood disadvantage or mental health problems, the explanatory power of these factors would been underestimated. The results from the sensitivity analysis comparing the complete and imputed datasets were comparable increasing confidence in our findings. That said, bias due to nonignorable attrition or a missing data-generating mechanism cannot be ruled out. Second, whilst we have characterised mental health problems as a confounder, these problems in adulthood were assessed at the same time as drug use, such that the attenuation we observed may be due to these factors acting as a mediator. Unfortunately, it was not possible to identify whether adult mental health problems were a confound or mediator as the periods covered by assessments of illicit drug use and mental health overlapped. Mediation is a less likely explanation for mental health problems in childhood as these assessments preceded the drug use assessment by 20 years. Third, our assessments of socioeconomic disadvantage and mental health may not have been sufficiently sensitive. Other measures that captured different aspects of childhood disadvantage, or a greater range of mental health symptoms, may have explained more variance in the illicit drug use-mortality association. Fourth, the assessments of illicit drugs omitted some benzodiazepines (e.g. alprazolam, diazepam) which is likely to have reduced prevalence and statistical power. The drug use assessments also did not include a measure of frequency of use or dose so we could not investigate the shape of the association (e.g. dose response or threshold) between drugs and mortality. Fifth, as we investigated the association between drug use in early adulthood and later mortality, deaths that occurred before early adulthood were omitted. The possibility of survivor bias is therefore a concern, where fewer participants who used drugs are available for analysis, such that the association with mortality might be underestimated. To explain the pattern of results we found this bias, would however, have to operate more for those exposed to adult than childhood socioeconomic disadvantage and also more on adult than maternal/ teacher reports of mental health problems.

## 5 Conclusion

In this first study to examine the explanatory power of socioeconomic disadvantage and mental health on the illicit drug use–mortality association, exposure to these factors in childhood did not help to explain this association. The same exposures in adulthood did not completely explain the drug use-mortality association, but, probably through a variety of mechanisms, likely contribute to generating it. Our findings, if causal, have implications for public health policy. The main is that as socioeconomic disadvantage and mental health problems in adulthood explained the excess mortality adding opportunities for employment (DeFulio et al., 2009), and effective treatments for mental health problems (van Dis et al., 2020) may help to enhance adult drug treatment.

## Funding

JW is funded by The Centre for the Development and Evaluation of Complex Interventions for Public Health Improvement (DECIPHer), a UKCRC Public Health Research Centre of Excellence. Joint funding (MR/KO232331/1) from the British Heart Foundation, Cancer Research UK, Economic and Social Research Council, Medical Research Council, the Welsh Government and the Wellcome Trust, under the auspices of the UK Clinical Research Collaboration, and is gratefully acknowledged. These funders had no influence on the analysis, decision to publish, or preparation of this manuscript.

## Author contributions

JW designed the study, acquired the data, undertook the analyses, and drafted the manuscript.

## Data availability

Data from the 1970 British Cohort Study is publicly available from the UK Data Archive. Further information on the procedures to obtain data from the 1970 British Cohort Study is described at: https://discover.ukdataservice.ac.uk/.

## Code availability

The code used to generate the results presented in the manuscript are available from the corresponding author on reasonable request.

## CRediT author statement

James White: Conceptualization, Methodology, Data curation, Investigation, Writing- Original draft preparation, Writing- Reviewing and Editing.

## Declaration of Competing Interest

The authors declare they have no conflict of interest.

## Appendix A Supplementary data

Supplementary material 1: figures and tables Image 1

Supplementary material 2 Image 2
