# Supplementary material for: Associations between illicit drug use in early adulthood and mortality: Findings from a National Birth Cohort
Source: Prev Med. 2022 Jun;159:107058. doi: 10.1016/j.ypmed.2022.107058 (PMC9227730; doi:10.1016/j.ypmed.2022.107058)
Supplement: Supplementary file 1 — Supplementary material 1: figures and tables [file mmc1.docx]

Born between 5th and 11th April 1970

(N = 17,287)

Enrolled at birth

(N = 16,571)

Stillborn and neonatal deaths

(N = 567)

Deaths between neonatal and 10 years

(N = 32)

Missing covariate data

(N = 5,171)

Survey at 10 years

(N = 14,874 interviewed)

Deaths between 10 and 30 years

(N = 152)

Missing illicit drug use

exposure data

(N = 164)

Missing covariate data

(N = 2,126)

Survey at 30 years

(N = 11,261 interviewed)

Mortality surveillance until 44 years

Date of death unknown

(N = 40)

Imputed analytical sample (N = 11,250)

Complete case sample

(N = 4,032)

**eFigure 1. Participation in the 1970 British Cohort Study**


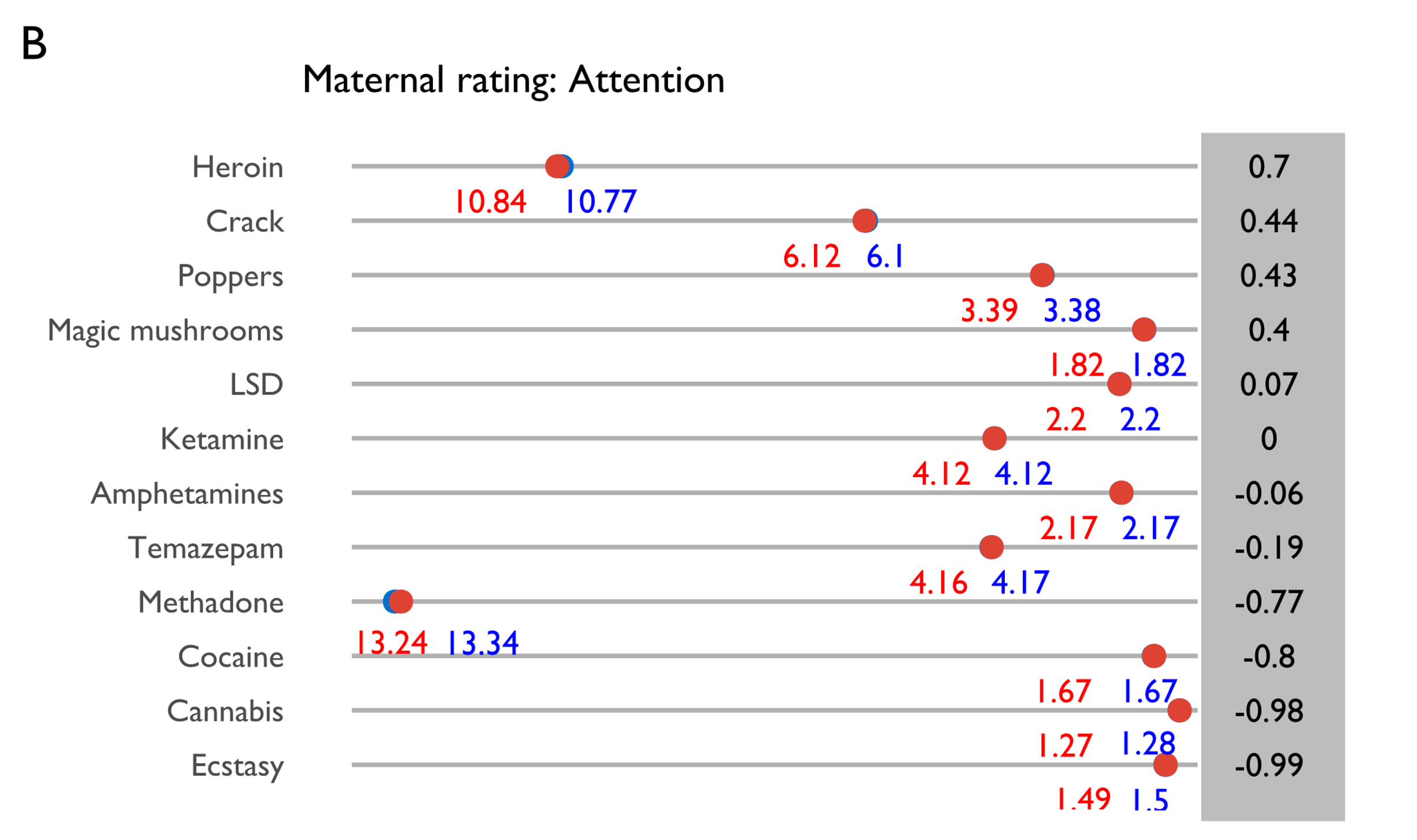

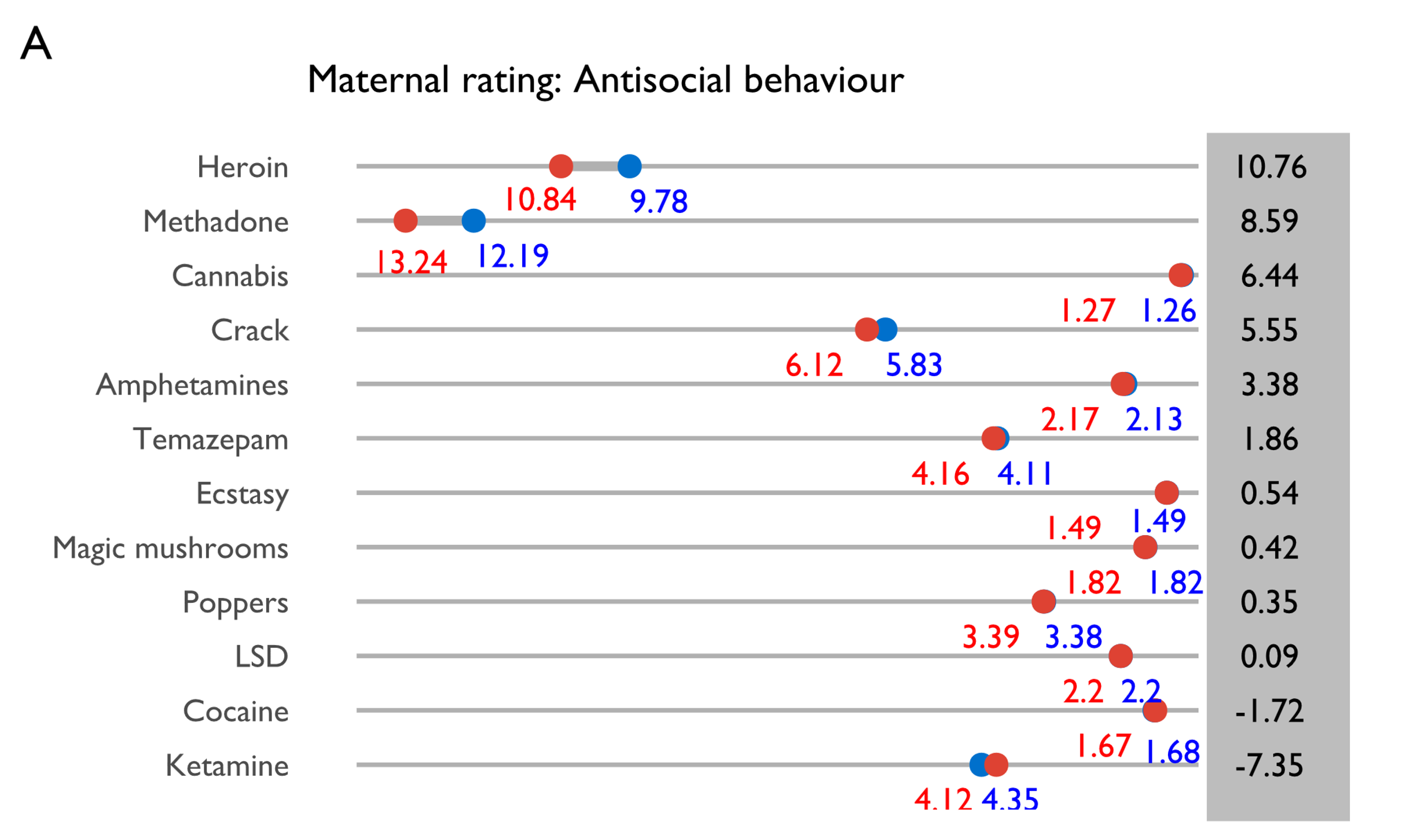


HR adjusted for sex plus symptoms/ behaviours

% Attenuation

HR adjusted for sex


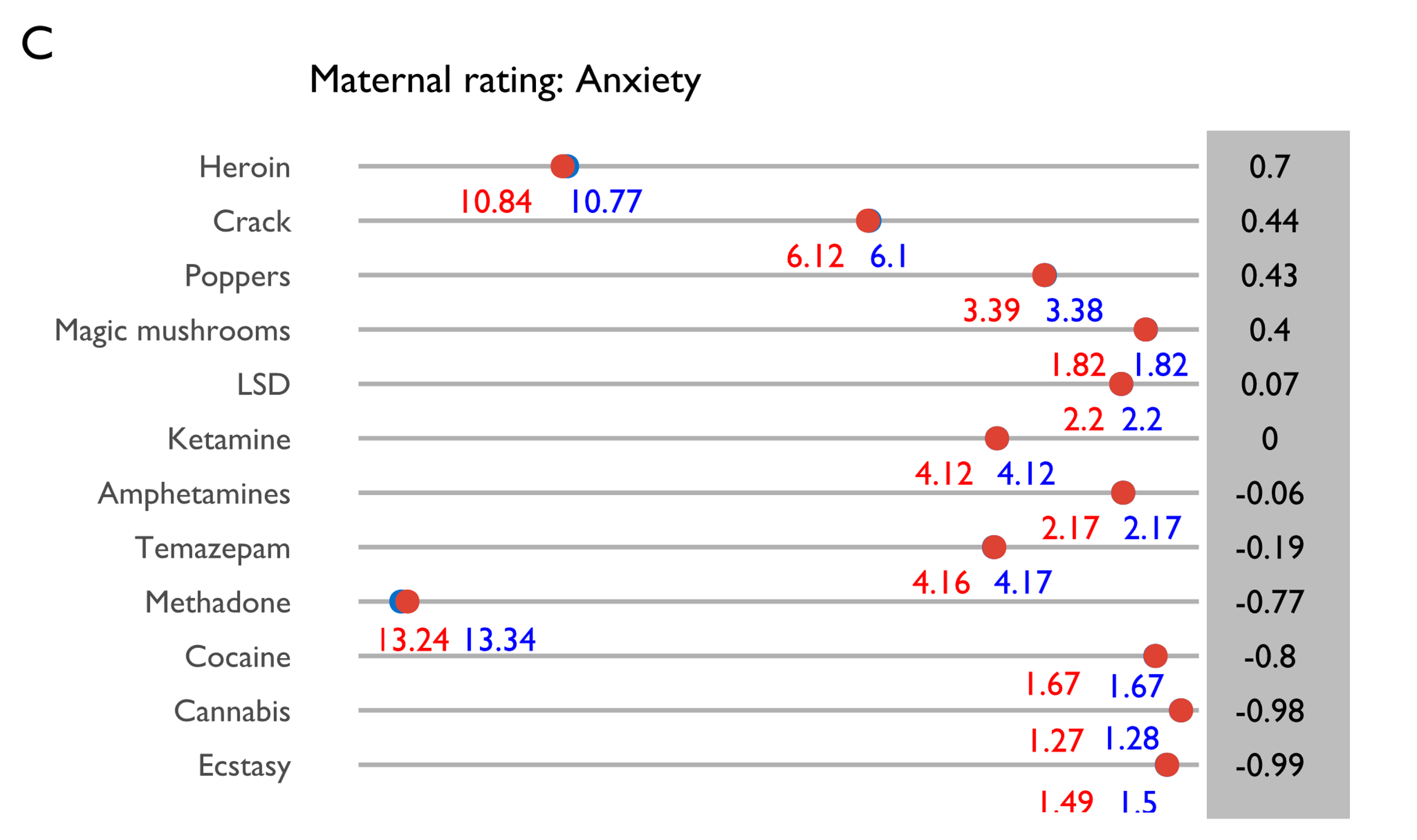


**eFigure 2. Dumbbell plot illustrating attenuation of illicit-drug mortality association by maternal assessment of child antisocial behaviour, attention problems and anxiety**

Notes: Grey box shows the percent attenuation using the formulae: ([HR sex-adjusted - 1]/ [HR covariate-adjusted - 1]/[HR sex-adjusted - 1]) / 100%. HR: hazard ratio.

**
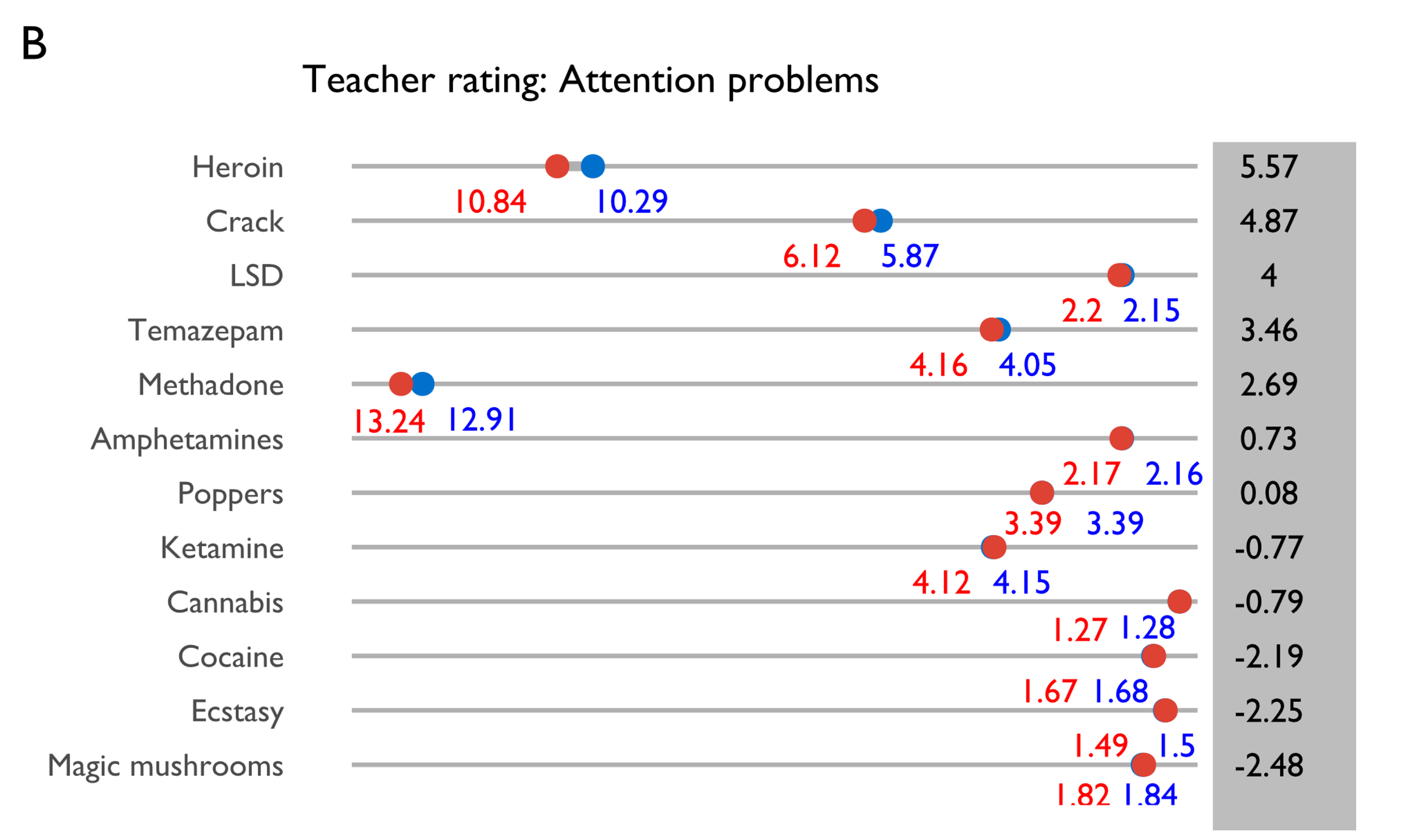

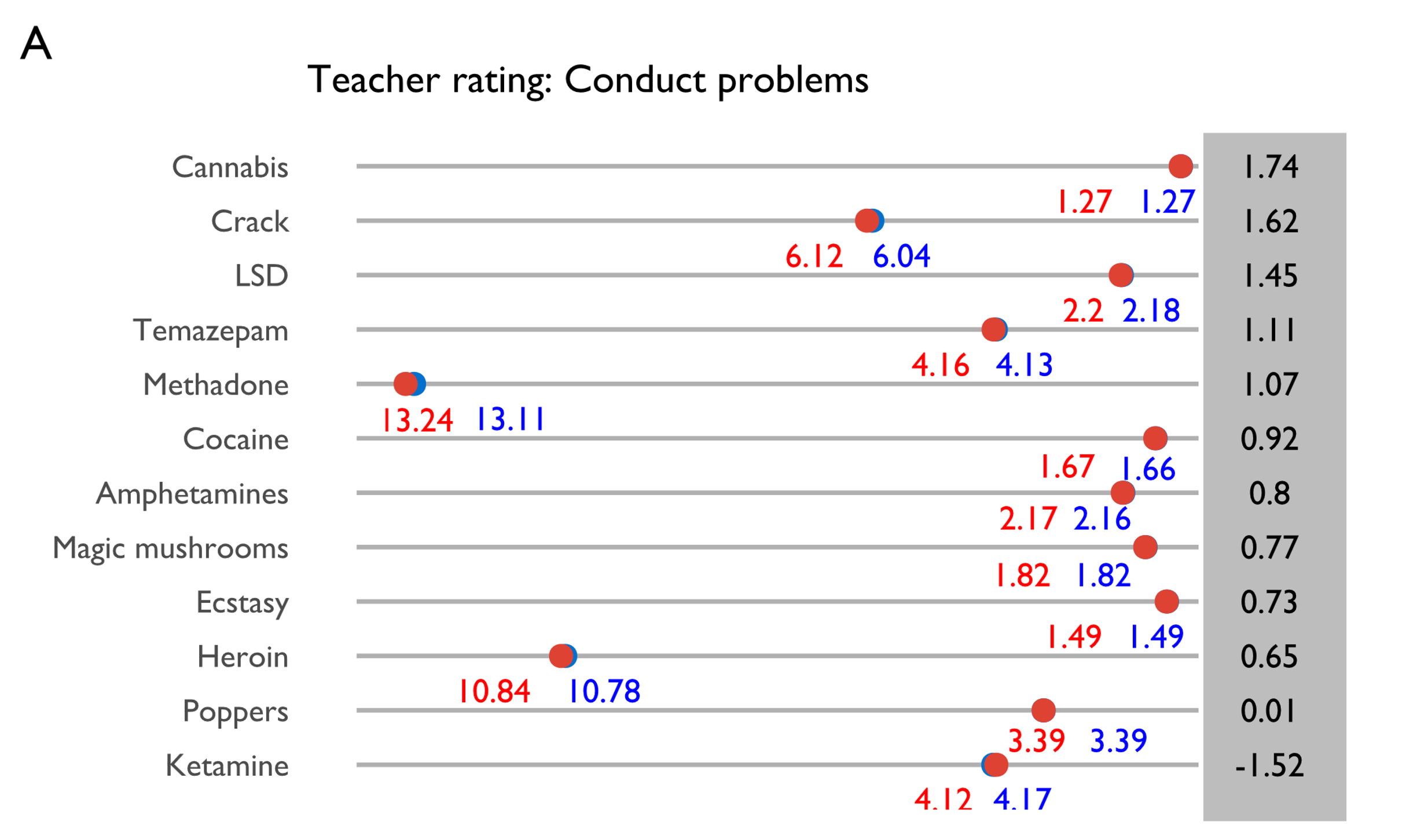
**

% Attenuation

HR adjusted for sex plus symptoms/ behaviours

HR adjusted for sex

**
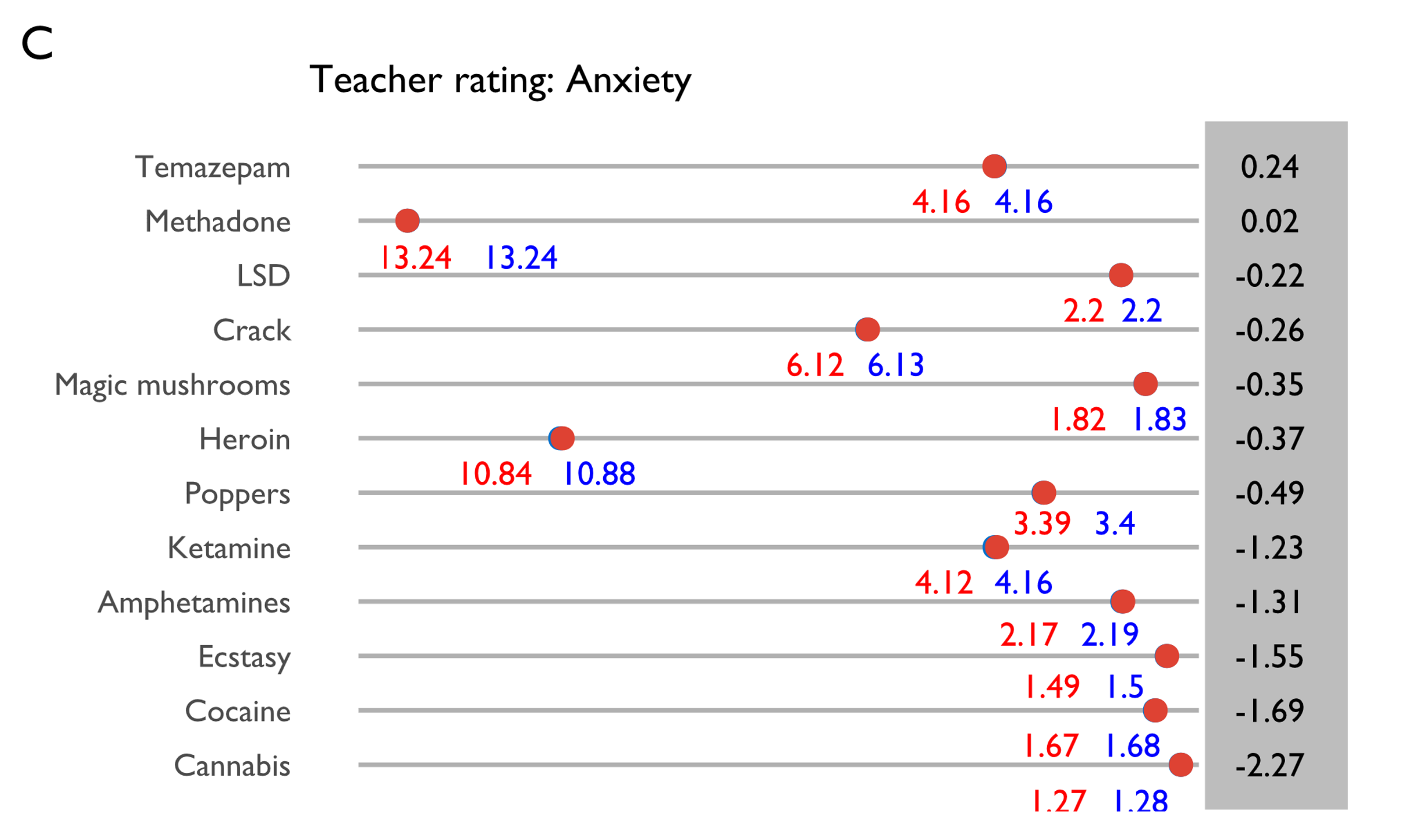
**

**eFigure 3. Dumbbell plot illustrating attenuation of illicit-drug mortality association by teacher ratings of childhood antisocial behaviours, attention problems and anxiety**

Notes: Grey box shows the percent attenuation using the formulae: ([HR sex-adjusted - 1]/ [HR covariate-adjusted - 1]/[HR sex-adjusted - 1]) / 100%. HR: hazard ratio.

**eTable 1. Association between participant characteristics with cannabis, stimulants and hallucinogen use in the past year (n = 11,250)**

|  | **Odds ratio (95% confidence interval)** | | | | | | |
| --- | --- | --- | --- | --- | --- | --- | --- |
|  | **Cannabis** | **Ecstasy** | **Amphetamines** | **Cocaine** | **Crack** | **LSD** | **Magic mushrooms** |
| Male | 2.32 (2.08, 2.60) | 2.66 (2.17, 3.25) | 2.66 (2.18, 3.26) | 2.46 (2.08, 2.92) | 4.71 (2.29, 9.67) | 3.20 (2.05, 5.00) | 2.80 (1.75, 4.50) |
| **Variables at 10-years ^a^** |  |  |  |  |  |  |  |
| Socioeconomic disadvantage |  |  |  |  |  |  |  |
| Lowest parental social class | 1.61 (1.15, 2.26) | 1.02 (0.57, 1.84) | 0.61 (0.34, 1.09) | 1.58 (1.93, 2.69) | 0.18 (0.02, 1.45) | 0.91 (0.29, 2.85) | 2.65 (0.56, 12.56) |
| Material circumstances | 0.98 (0.93, 1.03) | 1.01 (0.91, 1.12) | 1.04 (0.93, 1.15) | 0.88 (0.80, 0.97) | 1.38 (1.04, 1.82) | 0.97 (0.77, 1.22) | 1.04 (0.82, 1.31) |
| Family disruption | 1.25 (1.07, 1.46) | 1.58 (1.21, 2.06) | 1.54 (1.18, 2.01) | 1.30 (1.02, 1.66) | 1.50 (0.67, 3.34) | 1.71 (1.00, 2.96) | 2.02 (1.17, 3.48) |
| Mental health |  |  |  |  |  |  |  |
| Antisocial behaviour (maternal) | 1.09 (1.04, 1.15) | 1.05 (0.94, 1.16) | 1.14 (1.03, 1.25) | 1.00 (0.90, 1.09) | 1.26 (0.98, 1.61) | 1.08 (0.87, 1.33) | 1.02 (0.82, 1.27) |
| Anxiety (maternal) | 0.93 (0.88, 0.99) | 0.90 (0.80, 1.00) | 0.96 (0.86, 1.06) | 0.90 (0.82, 0.99) | 0.99 (0.73, 1.33) | 0.95 (0.75, 1.20) | 1.00 (0.79, 1.26) |
| Attention problem (maternal) | 1.09 (1.03, 1.14) | 1.13 (1.02, 1.24) | 1.15 (1.05, 1.27) | 1.06 (0.97, 1.15) | 1.30 (0.98, 1.73) | 1.10 (0.89, 1.37) | 1.00 (0.79, 1.25) |
| Antisocial behaviour (teacher) | 1.15 (1.08, 1.22) | 1.13 (1.00, 1.28) | 1.21 (1.08, 1.35) | 1.12 (1.02, 1.24) | 1.44 (1.00, 2.07) | 1.30 (1.05, 1.61) | 1.14 (0.89, 1.48) |
| Anxiety (teacher) | 0.85 (0.80, 0.91) | 0.85 (0.75, 0.97) | 0.84 (0.74, 0.96) | 0.81 (0.72, 0.90) | 0.83 (0.58, 1.20) | 0.93 (0.69, 1.25) | 0.84 (0.61, 1.15) |
| Attention problem (teacher) | 1.06 (0.99, 1.14) | 1.03 (0.91, 1.17) | 1.10 (0.98, 1.24) | 1.00 (0.91, 1.10) | 1.48 (1.09, 2.01) | 1.23 (0.93, 1.63) | 0.97 (0.74, 1.26) |
| Maternal psychological morbidity | 1.04 (0.98, 1.10) | 1.05 (0.95, 1.17) | 0.93 (0.85, 1.03) | 1.07 (0.97, 1.17) | 0.79 (0.60, 1.04) | 0.98 (0.79, 1.22) | 1.00 (0.80, 1.26) |
| **Variables at 30-years ^b^** |  |  |  |  |  |  |  |
| Socioeconomic disadvantage |  |  |  |  |  |  |  |
| Lowest occupational social class | 0.91 (0.71, 1.16) | 0.86 (0.53, 1.38) | 1.43 (0.80, 2.59) | 0.63 (0.42, 0.95) | 6.84 (0.92, 50.87) | 1.05 (0.31, 3.53) | 1.08 (0.32, 3.73) |
| No qualifications | 0.99 (0.88, 1.10) | 1.09 (0.89, 1.33) | 1.24 (1.02, 1.52) | 0.97 (0.81, 1.17) | 5.27 (2.92, 9.52) | 1.32 (0.87, 2.01) | 1.11 (0.69, 1.78) |
| Mental health |  |  |  |  |  |  |  |
| Psychiatric morbidity | 1.80 (1.58, 2.05) | 1.85 (1.47, 2.33) | 2.15 (1.73, 2.70) | 1.79 (1.64, 2.19) | 4.13 (2.29, 7.43) | 1.86 (1.16, 3.00) | 1.97 (1.18, 3.29) |
| Treatment for psychiatric problem | 1.67 (1.50, 1.86) | 1.82 (1.51, 2.21) | 1.97 (1.63, 2.38) | 1.44 (1.22, 1.71) | 4.11 (2.34, 7.24) | 2.35 (1.59, 3.47) | 1.67 (1.07, 2.60) |
| Alcohol problem (CAGE score ≥2) | 3.13 (2.76, 3.55) | 3.52 (2.87, 4.32) | 3.81 (3.11, 4.66) | 3.27 (2.73, 3.91) | 3.44 (1.89, 6.24) | 3.41 (2.25, 5.18) | 4.48 (2.89, 6.94) |
| Daily smoking | 4.92 (4.42, 5.48) | 4.41 (3.61, 5.40) | 5.44 (4.40, 6.72) | 4.42 (3.72, 5.26) | 19.28 (6.93, 53.62) | 3.92 (2.58, 5.95) | 6.64 (3.94, 11.19) |

**^a^** Occupational social class was paternal (maternal if paternal missing) using the Registrar General’s classification, material circumstances (sum of renting, household overcrowding (+1 person per room), receipt of state benefits, and no or shared access of either a bathroom, lavatory or hot water), and family disruption (any parental divorce, separation or death); Childhood mental health assessed antisocial behaviour, anxiety, and attention problems using the Rutter Parental ‘A’ Scale of Behaviour Disorder (maternal report) and the Social Development Scale (teacher report), and maternal psychological morbidity using Rutter’s Malaise Inventory. ^b^ Adult mental health comprises psychiatric morbidity (Malaise score ≥7) and having treatment for a psychiatric problem; Occupational social class using the Registrar General’s classification. LSD = Lysergic acid diethylamide.

**eTable 2. Association between childhood and adult characteristics with poppers, ketamine, temazepam and opiates (n = 11,250)**

|  | **Odds ratio (95% confidence interval)** | | | | |
| --- | --- | --- | --- | --- | --- |
|  | **Poppers** | **Temazepam** | **Ketamine** | **Heroin** | **Methadone** |
| Male | 3.35 (2.41, 4.67) | 1.50 (1.02, 2.22) | 3.98 (1.83, 8.69) | 4.83 (2.24, 10.39) | 2.69 (1.25, 5.80) |
| **Variables at 10-years ^a^** |  |  |  |  |  |
| Socioeconomic disadvantage |  |  |  |  |  |
| Lowest parental social class | 0.89 (0.36, 2.21) | 0.61 (0.15, 2.48) | 0.34 (0.03, 3.84) | 0.20 (0.06, 2.10) | 0.20 (0.02, 1.88) |
| Material circumstances | 0.96 (0.81, 1.13) | 1.09 (0.88, 1.34) | 1.00 (0.69, 1.45) | 1.28 (0.94, 1.73) | 1.46 (1.03, 2.05) |
| Family disruption | 1.05 (0.66, 1.68) | 1.46 (1.84, 2.53) | 1.79 (0.74, 4.34) | 1.98 (0.92, 4.28) | 1.66 (0.65, 4.32) |
| Mental health |  |  |  |  |  |
| Antisocial behaviour (maternal) | 1.08 (0.93, 1.26) | 1.07 (0.87, 1.30) | 0.79 (0.51, 1.23) | 1.62 (1.31, 1.99) | 1.52 (1.18, 1.96) |
| Anxiety (maternal) | 1.05 (0.90, 1.21) | 0.90 (0.72, 1.14) | 0.95 (0.67, 1.35) | 1.14 (0.83, 1.56) | 0.90 (0.61, 1.33) |
| Attention problem (maternal) | 1.09 (0.94, 1.27) | 1.16 (0.93, 1.43) | 1.10 (0.78, 1.55) | 1.60 (1.22, 2.10) | 1.26 (0.91, 1.76) |
| Antisocial behaviour (teacher) | 1.12 (0.93, 1.35) | 1.33 (1.10, 1.61) | 0.88 (0.52, 1.49) | 1.69 (1.25, 2.27) | 1.53 (1.05, 2.23) |
| Anxiety (teacher) | 0.89 (0.73, 1.08) | 1.03 (0.84, 1.29) | 0.67 (0.38, 1.17) | 0.96 (0.65, 1.41) | 0.90 (0.56, 1.44) |
| Attention problem (teacher) | 1.09 (0.92, 1.29) | 1.21 (0.98, 1.50) | 1.00 (0.64, 1.60) | 1.60 (1.14, 2.22) | 1.25 (0.83, 1.87) |
| Maternal psychological morbidity | 0.92 (0.79, 1.06) | 0.89 (0.73, 1.10) | 1.00 (0.69, 1.44) | 0.73 (0.56, 0.95) | 0.79 (0.55, 1.13) |
| **Variables at 30-years ^b^** |  |  |  |  |  |
| Socioeconomic disadvantage |  |  |  |  |  |
| Lowest occupational social class | 1.50 (0.60, 3.73) | 1.52 (1.13, 2.04) | 1.24 (0.73, 2.13) | 3.87 (1.86, 7.99) | 2.75 (1.00, 7.59) |
| No qualifications | 1.39 (1.02, 1.88) | 2.15 (1.45, 3.19) | 2.24 (1.18, 4.26) | 6.68 (3.48, 12.82) | 11.48 (4.66, 28.25) |
| Mental health |  |  |  |  |  |
| Psychiatric morbidity | 2.41 (1.73, 3.34) | 5.21 (3.52, 7.71) | 1.84 (0.84, 4.01) | 5.36 (2.96, 9.71) | 5.79 (2.86, 11.70) |
| Treatment for psychiatric problem | 2.41 (1.81, 3.22) | 6.67 (4.39, 10.13) | 2.98 (1.57, 5.64) | 10.24 (5.07, 20.63) | 12.63 (5.19, 30.71) |
| Alcohol problem (CAGE score ≥2) | 3.48 (2.56, 4.88) | 3.49 (2.31, 5.26) | 4.25 (2.20, 8.22) | 4.02 (2.17, 7.45) | 2.46 (1.10, 5.47) |
| Daily smoking | 3.60 (2.66, 4.88) | 4.48 (2.92, 6.89) | 4.21 (2.08, 8.50) | 13.27 (5.21, 33.83) | 11.73 (4.04, 34.08) |

**^a^** Occupational social class was paternal (maternal if paternal missing) using the Registrar General’s classification, material circumstances, and family disruption; Childhood mental health assessed antisocial behaviour, anxiety, and attention problems using the Rutter Parental ‘A’ Scale of Behaviour Disorder (maternal report) and the Social Development Scale (teacher report). ^b^ Adult mental health comprises psychiatric morbidity (Malaise score ≥7) and having treatment for a psychiatric problem; and maternal psychological morbidity using Rutter’s Malaise Inventory. Occupational social class using the Registrar General’s classification.

**eTable 3. Association of illicit drug use in the past year with all-cause mortality after adjusting for adult characteristics (n = 11,250)**

|  | **Hazard ratio (95% confidence interval)** | | | | | | |
| --- | --- | --- | --- | --- | --- | --- | --- |
|  | **Sex-adjusted** | **Adjusted for sex plus adult socioeconomic disadvantage ^a^** | **% change ^b^** | **Adjusted for sex plus adult mental health ^c^** | **% change** | **Adjusted for sex plus adult alcohol problems and daily smoking ^d^** | **% change** |
| **Any drug in past year** | 1.42 (0.95, 2.11) | 1.38 (0.92, 2.07) | 9.52 | 1.18 (0.79, 1.78) | 57.14 | 1.08 (0.70, 1.65) | 80.95 |
| **Cannabis** | 1.27 (0.83, 1.95) | 1.24 (0.80, 1.91) | 11.11 | 1.07 (0.69, 1.65) | 74.07 | 0.95 (0.61, 1.49) | 100 |
| **Ecstasy** | 1.49 (0.75, 2.95) | 1.53 (0.77, 3.03) | 8.16 | 1.23 (0.62, 2.45) | 53.06 | 1.11 (0.55, 2.22) | 77.55 |
| **Amphetamines** | 2.17 (1.19, 3.95) | 2.10 (1.15, 3.84) | 5.98 | 1.77 (0.96, 3.24) | 34.19 | 1.61 (0.86, 2.98) | 47.86 |
| **LSD** | 2.20 (0.70, 6.94) | 2.19 (0.69, 6.91) | 0.83 | 1.74 (0.55, 5.51) | 38.33 | 1.68 (0.53, 5.33) | 43.33 |
| **Magic mushrooms** | 1.82 (0.45, 7.37) | 1.86 (0.46, 7.54) | 4.88 | 1.49 (0.37, 6.04) | 40.24 | 1.26 (0.31, 5.16) | 68.29 |
| **Poppers** | 3.39 (1.65, 6.97) | 3.36 (1.63, 6.93) | 1.26 | 2.60 (1.25, 5.39) | 33.05 | 2.63 (1.27, 5.46) | 31.80 |
| **Cocaine** | 1.67 (0.94, 2.97) | 1.76 (0.98, 3.14) | 13.43 | 1.43 (0.80, 2.57) | 35.82 | 1.27 (0.70, 2.30) | 59.70 |
| **Temazepam** | 4.16 (1.70, 10.19) | 2.93 (1.07, 7.99) | 38.92 | 2.57 (1.03, 6.42) | 50.32 | 3.04 (1.23, 7.53) | 35.44 |
| **Ketamine** | 4.12 (1.02, 16.73) | 3.74 (0.92, 15.30) | 12.18 | 3.13 (0.77, 12.79) | 31.73 | 2.97 (0.73, 12.14) | 36.86 |
| **Crack** | 6.12 (2.25, 16.61) | 4.67 (1.68, 12.95) | 28.32 | 3.92 (1.42, 10.87) | 42.97 | 4.40 (1.60, 12.13) | 33.59 |
| **Heroin** | 10.84 (4.74, 24.79) | 7.87 (3.32, 18.67) | 30.18 | 6.38 (2.70, 15.07) | 45.33 | 7.66 (3.29, 17.85) | 32.32 |
| **Methadone** | 13.24 (5.39, 32.53) | 10.36 (4.06, 26.41) | 23.53 | 7.52 (2.97, 19.09) | 46.73 | 10.04 (4.03, 25.02) | 26.14 |

^a^ Adult socioeconomic disadvantage assessed at 30-years using the Registrar General’s classification and whether study members had any qualifications; ^b^ Based on a comparison of the sex-adjusted HR with that for the covariate-adjusted HR using the formulae: ([HR _sex-adjusted_ - 1]/ [HR _covariate-adjusted_ - 1]/[HR _sex-adjusted_ - 1]) / 100%; ^c^ Adult mental health comprises psychiatric morbidity (Malaise score ≥7) and having seen specialist for psychiatric problem; ^d^ Alcohol problems assessed as a CAGE score ≥2. LSD = Lysergic acid diethylamide.

**eTable 4. Association of illicit drug use in the past year with all-cause mortality in the complete case sample (n = 4,032)**

|  | **Hazard ratio (95% confidence interval)** | | | | | | |
| --- | --- | --- | --- | --- | --- | --- | --- |
|  | **Sex-adjusted** | **Adjusted for sex plus childhood socioeconomic disadvantage** ^b^ | **Adjusted for sex plus childhood mental health** ^b^ | **Adjusted for sex plus maternal psychological morbidity** | **Adjusted for sex plus adult socioeconomic disadvantage** ^c^ | **Adjusted for sex plus adult mental health** ^c^ | **Adjusted for sex plus adult alcohol problems and daily smoking** ^d^ |
| **Any drug in past year** | 1.28 (0.57, 2.84) | 1.29 (0.58, 2.88) | 1.25 (0.56, 2.79) | 1.28 (0.57, 2.84) | 1.10 (0.49, 2.48) | 1.28 (0.57, 2.85) | 1.11 (0.48, 2.58) |
| **Cannabis** | 0.82 (0.31, 2.13) | 0.83 (0.32, 2.16) | 0.80 (0.31, 2.09) | 0.82 (0.32, 2.13) | 0.70 (0.27, 1.85) | 0.82 (0.32, 2.14) | 0.68 (0.25, 3.57) |
| **Ecstasy** | 1.22 (0.29, 5.11) | 1.23 (0.29, 5.19) | 1.20 (0.29, 5.07) | 1.21 (0.29, 5.09) | 1.06 (0.25, 4.47) | 1.22 (0.29, 5.11) | 1.04 (0.24, 4.45) |
| **Amphetamines** | 1.99 (0.60, 6.55) | 2.04 (0.62, 6.72) | 1.93 (0.58, 6.40) | 1.99 (0.60, 6.57) | 1.72 (0.52, 5.72) | 1.92 (0.58, 6.34) | 1.69 (0.49, 5.81) |
| **LSD** | ^a^ | ^a^ | ^a^ | ^a^ | ^a^ | ^a^ | ^a^ |
| **Magic mushrooms** | ^a^ | ^a^ | ^a^ | ^a^ | ^a^ | ^a^ | ^a^ |
| **Poppers** | 5.35 (1.63, 17.62) | 4.97 (1.50, 16.46) | 5.35 (1.63, 17.62) | 5.42 (1.64, 17.87) | 4.56 (1.37, 15.15) | 5.46 (1.64, 18.13) | 4.84 (1.44, 16.31) |
| **Cocaine** | 2.08 (0.73, 5.93) | 2.09 (0.73, 5.96) | 2.02 (0.71, 5.79) | 2.07 (0.72, 5.90) | 1.82 (0.64, 5.24) | 2.24 (0.78, 6.42) | 1.86 (0.63, 5.49) |
| **Temazepam** | ^a^ | ^a^ | ^a^ | ^a^ | ^a^ | ^a^ | ^a^ |
| **Ketamine** | 10.31 (1.40, 75.84) | 9.01 (1.21, 67.29) | 9.92 (1.32, 74.39) | 10.32 (1.40, 75.93) | 10.37 (1.39, 77.10) | 9.65 (1.30, 71.87) | 7.50 (0.98, 57.55) |
| **Crack** | ^a^ | ^a^ | ^a^ | ^a^ | ^a^ | ^a^ | ^a^ |
| **Heroin** | ^a^ | ^a^ | ^a^ | ^a^ | ^a^ | ^a^ | ^a^ |
| **Methadone** | ^a^ | ^a^ | ^a^ | ^a^ | ^a^ | ^a^ | ^a^ |

a Unable to estimate due to too few deaths among users; ^b^ Childhood socioeconomic disadvantage comprises paternal (maternal if paternal missing) Registrar General’s classification, material circumstances, and family disruption; Childhood mental health assessed antisocial behaviour, anxiety, and attention problems using the Rutter Parental ‘A’ Scale of Behaviour Disorder (maternal report) and the Social Development Scale (teacher report); ^c^ Adult socioeconomic disadvantage assessed at 30-years using the Registrar General’s classification and whether study members had any qualifications; Adult mental health comprises psychiatric morbidity (Malaise score ≥7) and having seen specialist for psychiatric problem; ^d^ Alcohol problems assessed as a CAGE score ≥2. LSD = Lysergic acid diethylamide.

**eTable 5. Association of illicit drug use in the past year with all-cause mortality after adjusting for interactions between mental health problems and socioeconomic disadvantage in childhood and early adulthood (n = 11,250)**

|  | **Hazard ratio (95% confidence interval)** | | | | |
| --- | --- | --- | --- | --- | --- |
|  | **Sex-adjusted** | **Adjusted for sex plus childhood characteristics ^a^** | **% change ^b^** | **Adjusted for sex plus the adult characteristics ^c^** | **% change** |
| **Any drug in past year** | 1.42 (0.95, 2.11) | 1.43 (0.94, 2.16) | -2.38 | 1.15 (0.76, 1.74) | 64.29 |
| **Cannabis** | 1.27 (0.83, 1.95) | 1.26 (0.81, 1.97) | 3.70 | 1.04 (0.67, 1.61) | 85.19 |
| **Ecstasy** | 1.49 (0.75, 2.95) | 1.56 (0.77, 3.13) | -14.29 | 1.27 (0.64, 2.53) | 42.86 |
| **Amphetamines** | 2.17 (1.19, 3.95) | 2.19 (1.17, 4.08) | -1.71 | 1.72 (0.94, 3.17) | 35.90 |
| **LSD** | 2.20 (0.70, 6.94) | 2.14 (0.65, 6.95) | 5.00 | 1.71 (0.53, 5.47) | 35.83 |
| **Magic mushrooms** | 1.82 (0.45, 7.37) | 1.95 (0.47, 8.07) | -15.85 | 1.58 (0.38, 6.47) | 35.37 |
| **Poppers** | 3.39 (1.65, 6.97) | 3.49 (1.66, 7.37) | -4.18 | 2.57 (1.23, 5.36) | 31.80 |
| **Cocaine** | 1.67 (0.94, 2.97) | 1.69 (0.93, 3.10) | -2.99 | 1.53 (0.85, 2.76) | 22.39 |
| **Temazepam** | 4.16 (1.70, 10.19) | 4.21 (1.64, 10.79) | -1.58 | 1.88 (0.67, 5.31) | 72.15 |
| **Ketamine** | 4.12 (1.02, 16.73) | 4.49 (1.03, 19.38) | -11.86 | 2.73 (0.66, 11.39) | 40.38 |
| **Crack** | 6.12 (2.25, 16.61) | 4.83 (1.53, 15.26) | 25.20 | 3.16 (1.10, 9.03) | 58.20 |
| **Heroin** | 10.84 (4.74, 24.79) | 9.14 (3.40, 24.47) | 17.28 | 5.14 (2.05, 12.90) | 59.15 |
| **Methadone** | 13.24 (5.39, 32.53) | 12.36 (3.98, 38.37) | 7.19 | 6.79 (2.50, 18.42) | 54.66 |

^a^ Childhood environment comprises paternal (maternal if paternal missing) Registrar General’s classification, material circumstances and family disruption, childhood mental health problems with antisocial behaviour, anxiety, and attention assessed by mothers and teachers, plus maternal psychological morbidity. ^b^ Based on a comparison of the sex-adjusted HR with that for the covariate-adjusted HR using the formulae: ([HR sex-adjusted - 1]/ [HR covariate-adjusted - 1]/[HR sex-adjusted - 1]) / 100%. ^c^ Adult environment comprises psychiatric morbidity (Malaise score ≥7) and having seen specialist for psychiatric problem, socioeconomic disadvantage assessed at 30-years using the Registrar General’s classification and whether study members had any qualifications.

LSD = Lysergic acid diethylamide

**eTable 6. Association of illicit drug use in the past year with all-cause mortality with and without removing opiate users**

|  | **Hazard ratio (95% confidence interval)** | |
| --- | --- | --- |
|  | **Sex-adjusted** | **Adjusted for sex after removal of opiate users** |
| **Any drug in past year** | 1.42 (0.95, 2.11) | 1.08 (0.70, 1.66) |
| **Cannabis** | 1.27 (0.83, 1.95) | 0.99 (0.62, 1.58) |
| **Ecstasy** | 1.49 (0.75, 2.95) | 1.28 (0.59, 2.74) |
| **Amphetamines** | 2.17 (1.19, 3.95) | 1.54 (0.75, 3.17) |
| **LSD** | 2.20 (0.70, 6.94) | 0.83 (0.12, 5.95) |
| **Magic mushrooms** | 1.82 (0.45, 7.37) | 1.09 (0.15, 7.83) |
| **Poppers** | 3.39 (1.65, 6.97) | 2.85 (1.25, 6.51) |
| **Cocaine** | 1.67 (0.94, 2.97) | 1.23 (0.62, 2.43) |
| **Temazepam** | 4.16 (1.70, 10.19) | 3.38 (1.07, 10.65) |
| **Ketamine** | 4.12 (1.02, 16.73) | 2.90 (0.40, 20.84) |
| **Crack** | 6.12 (2.25, 16.61) | 4.01 (0.56, 28.58) |

LSD = Lysergic acid diethylamide. Opiate users comprise those who have used methadone or heroin in the past month.
